# Retroperitoneal leiomyosarcoma mimicking gastric cancer recurrence: A case report

**DOI:** 10.1016/j.ijscr.2019.10.028

**Published:** 2019-10-21

**Authors:** Woo Yong Lee, Hye Kyeong Lee

**Affiliations:** a Department of Surgery, Seoul Paik Hospital, Inje University College of Medicine, 9, Mareunnae-ro, Jung-gu, Seoul 100-032, Republic of Korea; b Department of Pathology, Seoul Paik Hospital, Inje University College of Medicine, 9, Mareunnae-ro, Jung-gu, Seoul 100-032, Republic of Korea

**Keywords:** Gastric cancer, Local recurrence, Leiomyosarcoma

## Abstract

**Introduction:**

Leiomyosarcoma is a rare soft tissue cancer that arises from smooth muscle cells that form involuntary muscles. Here, we report a rare case of retroperitoneal leiomyosarcoma that mimicked a metastatic tumor arising from a gastric cancer.

**Presentation of case:**

A 43-year-old man underwent radical total gastrectomy for gastric cancer. He underwent adjuvant chemotherapy with oral 5-fluorouracil (5-FU) for 2 years and regular follow-up. Twenty-nine months after gastrectomy, computed tomography (CT) was performed and revealed a solitary localized mass measuring 43 mm in diameter among retroperitoneal lymph nodes of the posterior inferior vena cava. This finding indicated that the previous gastric cancer recurred either in the lymph node or retroperitoneum. Second-line chemotherapy, consisting of oxaliplatin and 5-FU (FOLFOX) was administered in four cycles. However, serial CT scans showed increased tumor size. Retroperitoneal tumor did not respond to treatment, surgical resection was performed. Pathological findings led to a diagnosis of pleomorphic and epithelioid leiomyosarcoma without metastatic adenocarcinoma of the stomach.

**Discussion:**

When a diagnosis of locoregional recurrence of gastric cancer in the retroperitoneum is considered, retroperitoneal leiomyosarcoma should also be considered in the differential diagnosis.

**Conclusion:**

Early diagnosis and concerted therapeutic efforts are important for managing misdiagnosed metastatic gastric cancer.

## Highlights

• Leiomyosarcoma is a rare soft tissue cancer.
• Retroperitoneal tumors must be ruled out if the tumor is suspected to be a regional recurrence.
• Early diagnosis and concerted therapeutic efforts are important for managing misdiagnosed metastatic gastric cancer.

## 1 Introduction

Leiomyosarcoma is a rare soft tissue cancer that arises from smooth muscle cells that form the involuntary muscles [1]. It usually develops in the uterus, in the gastrointestinal tract, and rarely in skeletal tissues; however, it frequently occurs in the retroperitoneum, and leiomyosarcomas and liposarcomas are the most common types of sarcoma [2].

Only a few cases of retroperitoneal leiomyosarcoma that mimic gastric cancer recurrence after gastrectomy have been reported. Herein, we report a rare case of retroperitoneal leiomyosarcoma that mimicked a metastatic tumor arising from gastric cancer. This paper has been reported in line with the SCARE criteria [3].

## 2 Case presentation

**Fig. 1:**
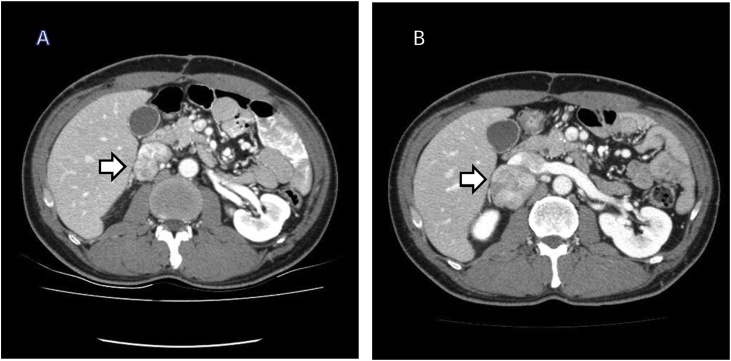
Computed tomography scan. Computed tomography scans showed an enlarged retroperitoneal lymph node of the posterior inferior vena cava (A) and rapidly enlarged lymph node after chemotherapy (B).

**Fig. 2:**
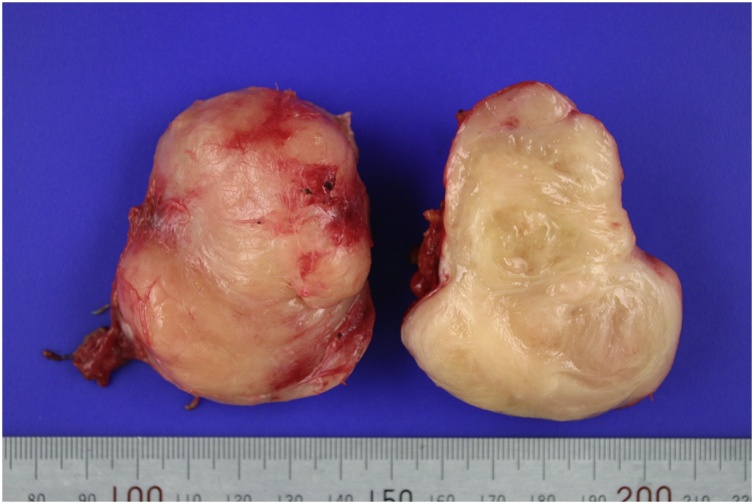
Macroscopic finding. Grossly, the tumor was hard and fibrotic, measuring 66 × 58 × 50 mm. The cut surfaces were multinodular, diffusely edematous, and myxoid with focal petechial hemorrhagic necrosis (approximately 15%) and fish-flesh appearance.

**Fig. 3:**
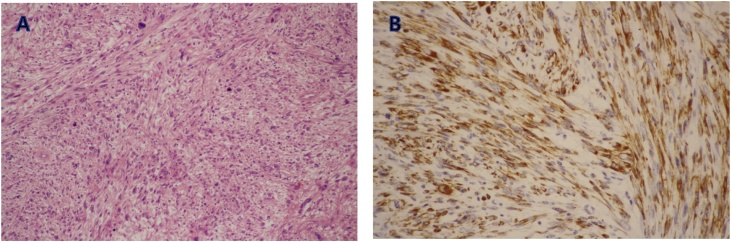
Histological examination of biopsy. (A) pleomorphism with bizarre multinucleated tumor cells and high frequency mitosis (hematoxylin and eosin, ×100). (B) Tumor cells showed strong reactivity for desmin (desmin, ×200).

A 43-year-old man underwent radical total gastrectomy for gastric cancer. He was not taking medications for specific diseases such as hypertension or diabetes, and had no other familial, environmental, or social problems. A postoperative intra-abdominal abscess developed around the anastomotic site due to an esophagojejunal anastomotic leak, which improved after percutaneous drainage and conservative therapy. Histopathological examination of the tumor revealed mixed differentiated adenocarcinoma; the tumor was classified as stage IB (pT1bN1M0) according to the 7th TNM classification of the International Union Against Cancer. He received adjuvant chemotherapy with oral 5-FU for 2 years. Analysis of tumor marker levels (CEA and CA19-9) was performed every 3 months and computed tomography (CT) imaging and gastrofibroscopy (GFS) every 6 months. About 29 months after gastrectomy, CT imaging was performed and revealed a solitary localized mass measuring 43 mm in diameter among the retroperitoneal lymph nodes of the posterior inferior vena cava (IVC) (Fig. 1A). The patient’s physical examination, neurological examination, laboratory including tumor marker analysis, and other radiologic findings (including positron emission tomography-CT) were unremarkable. We considered that his previous gastric cancer recurred either in a lymph node or the retroperitoneum. Second-line chemotherapy consisting of oxaliplatin and 5-FU (FOLFOX) was administered in four cycles. However, a follow-up CT scan indicated that the tumor rapidly enlarged (59 mm); no new lesions were observed (Fig. 1B). Hence, we decided to completely resect the tumor due to lack of response to the chemotherapy. His vital signs and laboratory findings upon admission were normal, including CEA level (1.9 ng/mL) and CA19-9 level (10.6 U/mL). His operative finding was the tumor strongly adhered to the IVC; therefore, this part of the IVC was also removed via an en bloc resection of the tumor mass. Ascites and peritoneal disseminated lesions were not detected during the procedure. Macroscopically, the tumor was hard and fibrotic, measuring 66 × 58 × 50 mm. The cut surfaces were multinodular, diffusely edematous, and myxoid with focal petechial hemorrhagic necrosis (approximately 15%) and had a fish-flesh appearance (Fig. 2). The pathological examination showed pleomorphism with bizarre multinucleated tumor cells and an average of 19 mitoses per 10 high-power fields in the tumors (Fig. 3A). The tumor was immunohistochemically positive for smooth muscle actin (SMA), desmin, and H-caldesmon and negative for c-KIT, CD34, and S-100 (Fig. 3B).

Finally, the patient was diagnosed with pleomorphic and epithelioid leiomyosarcoma with high mitotic activity. We assumed that the leiomyosarcoma was a second primary malignancy that developed from the retroperitoneum.

The patient’s postoperative course was favorable. He received adjuvant chemotherapy and radiotherapy. He did not experience disease recurrence for 3 years and underwent surgical resection of locoregional and lung metastasis. He survived for 5 years.

## 3 Discussion

Retroperitoneal leiomyosarcoma is a rare malignant cancer that develops in the soft tissue of the retroperitoneal cavity. Leiomyosarcoma accounts for approximately 50% of retroperitoneal tumors and can also arise directly from the viscera, including the gastrointestinal tract and uterus [4]. Leiomyosarcoma mainly occurs during the sixth decade of life and is common in women [5]. This tumor usually presents without associated symptoms; however, symptoms may occur as the tumor increases in size. For example, tumors >10 cm in diameter can present with abdominal pain, abdominal fullness, or neurologic symptoms [6].This was a case of a patient with retroperitoneal leiomyosarcoma that was misdiagnosed as a metastatic lymph node and had a delayed diagnosis.

Gastric cancer recurrence is classified as locoregional, peritoneal seeding, hematogenous, and multiple metastasis [7]. Locoregional metastases include dominant masses in the gastric bed, upper abdominal retroperitoneal lymph nodes, or anastomotic recurrence. The geographic differences in patterns of recurrence were noted. Specifically, individuals living in Eastern countries had a lower incidence of locoregional recurrence than those living in Western countries. Hartgrink et al. reported that locoregional recurrence remains a significant problem with an incidence of 32%–42% [8]. Although the results vary slightly, early detection of recurrence is very important. Because gastric cancer has various patterns of recurrence, close follow-up is important for early detection of recurrence, and subsequent tailored treatment should be provided as soon as possible. Despite poor survival after recurrence of gastric cancer and unfavorable responses to chemotherapy in some patients, other patients have demonstrated partial or even complete response to chemotherapy and had long-term survival.

The pathological diagnosis of retroperitoneal nodules by aspiration biopsy is difficult and can cause complications such as infection, metastasis, or hemorrhage in the abdominal cavity. In this report, the patient was initially managed with chemotherapy but follow-up imaging showed an increase in tumor size despite treatment. At that point, the tumor was completely resected for treatment and diagnosis. The patient was finally diagnosed with retroperitoneal leiomyosarcoma and then received appropriate chemotherapy and radiation for treatment.

The most crucial treatment for retroperitoneal leiomyosarcoma is complete surgical excision. Prognosis depends on complete removal of the tumor without any complications such as perforation. Most studies have shown local recurrence within 3 years after excision of the primary mass. Zhang et al. [9] reported that approximately 80%–87% of all local recurrences become evident within 2 years and 100% are detected within 3 years. In this case report, local recurrence was treated with chemotherapy and radiation.

## 4 Conclusion

We suggest that retroperitoneal tumors must be ruled out even if the tumor is highly suspected to be a locoregional recurrence. When a locoregional recurrence of gastric cancer in the retroperitoneum is considered, leiomyosarcoma should also be considered in the differential diagnosis. Early diagnosis and concerted therapeutic efforts are important for managing misdiagnosed metastatic gastric cancer.

## Sources of funding

This study did not receive any funding support.

## Ethical approval

This is a case report; therefore it did not require ethical approval from ethics committee.

## Consent

Informed written consent was obtained from the patient for publication of this report and any accompanying images.

## Author contribution

Lee WY and Lee HK were attending doctors for the patient. Lee WY performed the surgical operation and Lee HK prepared the pathologic report. Lee WY and Lee HK organized the report and wrote the paper. All authors were involved in drafting and revising the manuscript, and all authors read and approved the final manuscript.

## Registration of research studies

Not applicable.

## Guarantor

Woo Yong Lee.

## Provenance and peer review

Not commissioned, externally peer-reviewed.

## Declaration of Competing Interest

The authors declare that we have no conflict of interest.
